# Mycotoxins in Basidiomycete Mycelium and Fruiting Bodies: Implications for Eu Novel Food Assessment and Selected International Regulatory Frameworks

**DOI:** 10.1111/1541-4337.70614

**Published:** 2026-08-23

**Authors:** Niklas Erik Wilhelm Berglund, Pauliina Vappu Lankinen, Kirsi Susanna Mikkonen

**Affiliations:** ^1^ Department of Food and Nutrition Faculty of Agriculture and Forestry University of Helsinki Helsinki Finland; ^2^ Department of Microbiology Faculty of Agriculture and Forestry University of Helsinki Helsinki Finland; ^3^ Helsinki Institute of Sustainability Science University of Helsinki Helsinki Finland

## Abstract

Advances in technology have enabled the efficient and sustainable production of basidiomycete vegetative mycelium, particularly from *Pleurotus* species, as a novel food source. However, mycelium has yet to receive regulatory approval in many global markets, requiring a better understanding of factors affecting its safety. Yet, various studies on different species and mycotoxins suggest that mycelium may be safer than its fruiting body. This review explores the occurrence and distribution of basidiomycete mycotoxins (BMTs) across different species and fungal structures, with a particular focus on *Pleurotus* spp., given their prominent role in mycelium‐based products. BMTs are less studied compared to the well‐regulated ascomycete toxins. The authors reviewed all available literature on food‑safety‑relevant BMTs. These include, but are not limited to, amatoxins, coprine, illudins, muscarine, ostreolysin, and psilocybin. The toxins were identified from a resource developed for the European Food Safety Authority's novel food safety assessment. The main finding was that BMTs are predominantly present in the fruiting body rather than in mycelium. Regardless of species or BMT, mycelium showed lower levels compared to the fruiting body. Notably, no mycotoxins exclusive to the mycelium were identified, whereas certain BMTs detected in fruiting bodies were absent in mycelium. This review, the first of its kind, highlights the potential of basidiomycete mycelium as a novel and sustainable food. It also identifies gaps in toxicological data, aiming to inform regulatory bodies and support the development of novel fungal‐based foods and materials.

## 1 Introduction

Fungi have been a part of the human diet since prehistoric times (Berger et al. 2022). Edible fungi generally enjoy a positive perception among consumers largely due to characteristic flavor and consistency, the assumption of sustainable origin, and low caloric content along with presumed health benefits (Predanócyová et al. 2023).

Over 200 genera of fungi are considered safe for consumption (Brainerd 2013). Historically, the majority of edible fungi have been consumed as fruiting bodies of basidiomycetes and single‐cell (yeast) or filamentous (e.g., mold) ascomycetes (Berger et al. 2022). However, the vegetative mycelium of basidiomycetes, the fungal division best known for its species’ fruiting bodies that are commonly referred to as mushrooms, has not been approved for use as food by regulatory authorities in most major markets, such as China and the European Union (NHC 2023; European Commission 2026). Although basidiomycete mycelium has been considered suitable for food use in the United States since 1988, a recent Generally Recognized as Safe (GRAS) notification was withdrawn by the applicant, citing a lack of sufficient safety data (FDA 1988, 2024c). The cautious regulatory stance contrasts with strong evidence that mycelium may be at least as safe as, or even safer than, the respective fruiting bodies. Elaborating on this topic is the central focus of this paper.

Basidiomycete vegetative mycelium, hereafter referred to as mycelium, is a thread‐like structure consisting of fused cell filaments that expand to collect nutrients from the surrounding growth medium. The idea of harvesting mycelium for food use has only emerged in recent times (Berger et al. 2022). Mycelium cultivation has several advantages compared with fruiting body cultivation. Because mycelial growth is independent of season, location, and external triggering signals, it can occur continuously rather than in intermittent flushes (Berger et al. 2022). Compared with plant‐ and animal‐based protein sources, mycelium cultivation requires up to 2 m^2^ less land use per kilogram of protein, can be cultivated vertically to further reduce land usage, uses roughly half the water needed for plant protein sources, and can rely on a wide range of low‑cost agri‑food side streams as growth substrates (Benetto et al. 2018).

Basidiomycete mycelium lacks a documented history of human consumption, which is why novel food or equivalent authorization processes are required in various global markets before it can be sold to consumers. The authorization processes range from literature reviews to clinical toxicology depending on the market. However, despite the different requirements of the regulators, evidence supporting mycelium's safety is beyond anecdotal, both for toxic and nontoxic species. Kaya et al. (2015) showed that the mycelium of *Amanita phalloides*, one of the world's most toxic fungi, contains toxin levels that are 30–100 times lower than those found in its fruiting body. While, for example, *Pleurotus* spp. fruiting bodies are generally considered safe for consumption, they contain pH‐ and heat‐sensitive toxins with hemo‐ and cytolytic potential such as OlyA/PlyB (ostreolysin A and pleurotolysin B) and ostreatin. Studies indicate that these mycotoxins are primarily produced in the fruiting bodies, making *Pleurotus* spp. mycelium comparatively safer (Berne et al. 2007). However, these compounds are typically inactivated during cooking and digestion and therefore do not pose a major health risk (Berne et al. 2007).

Given that mycelium serves different functions and exhibits varied metabolic processes in fungi, the general legislative standpoint is that its safety should not be assumed from fruiting bodies. This is emphasized, for example, in the following consultation statement by the European Parliament and the Council on the novel food status of *Coprinus comatus* mycelium “although the carpophore (reproductive part) has a history of safe and significant consumption in the European Union before May 15, 1997, this consumption history is not valid for the mycelium (vegetative part), and a relationship of equivalence between both parts at the level of nutritional composition, metabolites and potentially active substances has not been established.” (European Commission 2019).

The toxicity mechanisms in basidiomycete mycelium remain largely unknown, and to date, no publicly accessible review article has addressed this topic. Furthermore, most studies on basidiomycete mycotoxins to date are follow‑up investigations of poisoning incidents involving fruiting bodies rather than mycelium. This review article aims to compile the most recent and relevant studies to identify the occurrence and distribution of basidiomycete mycotoxins. *Pleurotus* spp. constitute the largest share of basidiomycete mycelium‐based food innovations and are therefore given particular emphasis. This is evidenced, for example, by products already sold on the US market, for example, MyBacon (MyFOREST Foods, New York, US), and the number of consultation requests for novel food status in the EU (European Commission 2019). This paper seeks to determine the taxonomic distribution of the most relevant mycotoxins and will highlight any observed similarities and differences in toxin profiles between fruiting bodies and mycelium.

## 2 Current Regulatory Framework for Mycelium and Basidiomycete Mycotoxins

The challenges in introducing mycelium products arise from the novelty of both the production methods and fungal structures. For example, in the EU, to gain market authorization for a novel food, a product must undergo a thorough evaluation by the European Food Safety Authority (EFSA) and the European Commission. Mycelium falls under the EU regulation (EU) 2015/2283, which defines novel food as “any food that was not used for human consumption to a significant degree within the Union before May 15, 1997”, including “food consisting of, isolated from or produced from microorganisms, fungi, or algae” and “food resulting from a production process not used for food production within the Union before May 15, 1997” (European Union. n.d.‐a).

The lack of consumption history and the limited number of safety studies conducted to date have not been sufficient to meet regulatory approval requirements for basidiomycete mycelium. Thus, it would need novel food or equivalent authorization processes in most markets, including the EU, China (New Food Raw Material), and Singapore. An exception is the United States, where it is optional, but worthwhile in terms of quality assurance, to petition for GRAS designation for new ingredients. Previously, companies have been able to independently determine that a substance is GRAS without FDA review. However, the FDA has announced plans to propose a regulation in 2026 to “require the submission to FDA of GRAS notices for all new substances claimed to be GRAS,” which, if implemented, would end the current self‐GRAS framework (FDA 2026).

While the future regulatory developments in the United States are unclear, the legislation has only become stricter in the EU, where the Qualified Presumption of Safety (QPS) status of filamentous fungi, including basidiomycetes, was removed in 2020 (EFSA 2020a). QPS is a simplified safety assessment used by the EFSA for microorganisms. QPS allows, for example, the deduction of safety information from related and authorized species.

With the increase in regulation in the United States and EU, the importance of safety studies of basidiomycete mycelium will only increase in the future. In the European Union, no basidiomycete mycelium has been authorized for food use. However, pea and rice protein fermented by *Lentinula edodes* mycelium has been authorized, but the mycelium as a processing aid (EFSA 2022). Still, what is remarkable in this case is that EFSA waived the requirement for toxicological studies, a decision that is unprecedented. The product holds GRAS “without further questions” status in the United States, which could have influenced EFSA's assessment.

Mycelium has been considered suitable for food use in the United States since 1976, and basidiomycete mycelium foods are actively being sold to customers, for example, the *Pleurotus ostreatus* mycelium product MyBacon (MyFOREST Foods, New York, US) (FDA 1988). Yet, some manufacturers have applied for GRAS designation for their products, for example, beta‐glucan from *Ganoderma lucidum* mycelium already holds this title.

In contrast, a case in 2024 involved the FDA ceasing its evaluation of *Pleurotus pulmonarius* mycelium at the notifier's request, citing safety considerations (FDA 2024c). Similarly, in August 2025, the German authorities confirmed that *P. pulmonarius* mycelium falls under the scope of the EU Novel Food Regulation (EU) 2015/2283 and therefore requires a full Novel Food safety assessment and authorization before market placement. However, in March 2026, *P. pulmonarius* mycelium was approved for sale in Singapore, making it the first basidiomycete mycelium in a major market to receive explicit regulatory authorization (SFA 2026).

*L. edodes* mycelium is widely approved worldwide for use as a processing aid, as previously mentioned in the EU. However, this designation requires that the mycelium itself be removed following processing. Outside of the previously mentioned markets, it is authorized in South American countries like Brazil and Chile, and Asian countries like India, Indonesia, Japan, the Philippines, South Korea, Thailand, and Singapore (Food Standards Australia New Zealand 2023).

In China, derivatives of *Hericium erinaceus* (lion's mane) have been authorized for medicinal use (He et al. 2017). In 2023, the Chinese National Health Commission rejected an application for New Food Raw Material approval for *Pleurotus eryngii* mycelium (NHC 2023). The reason remains unknown to the public.

In the EU, EFSA requires applicants to submit a comprehensive dossier containing, among other things, toxicological data (EFSA 2024). The toxicological data should compile information on potential harmful effects, for example, mycotoxins, contaminants, and heavy metals. This is intended to help the administration conduct literature reviews for risk assessment. Certain ascomycete toxins (e.g., Ochratoxin A, deoxynivalenol) are regulated in the EU, but they are not produced by basidiomycetes (van Dam et al. 2024). Thus, only basidiomycete mycotoxins are addressed in this review article. EFSA has not published an official list of harmful compounds to consider in the dossier; however, it has published a *Systematic Literature Review for EFSA Novel Food Safety Assessment* (EFSA 2020b) with the aim of developing a standard operating procedure for screening literature for novel food requests based on pre‐defined inclusion and exclusion criteria. The resource contains an exhaustive list of relevant harmful compounds for algae, chemicals, fungi, plants, and insects. However, the resource does not represent the official position of EFSA but was deemed successful by the authority when used in reviewing 36 Novel Food requests. All basidiomycete mycotoxins identified in the document will be addressed in this review, with some in more detail.

The FDA, on the other hand, places the responsibility on manufacturers to provide sufficient safety information for proposed food ingredients. While additional details may be required to satisfy the FDA, there is no specific list of harmful compounds that applicants must address. Additionally, the FDA only monitors certain ascomycete toxins in foods; basidiomycete toxins are not monitored (FDA 2024a).

China is similar to the other jurisdictions in this sense, since China only has guidelines and maximum levels set for ascomycete toxins (USDA 2018).

In conclusion, the global regulatory landscape for basidiomycete mycotoxins and mycelium remains in its early stages. Instead of easing application processes for mycelium products, regulatory authorities in China, the EU, and the United States have either rejected applications or imposed stricter requirements. What is clear is that rigorous risk assessment processes will remain in place, thereby maintaining the importance of comprehensive safety studies.

## 3 *Pleurotus* spp

*Pleurotus* is a genus with up to 70 species (OECD 2015). The type species is *P. ostreatus*, the oyster mushroom, which also is the most cultivated species within its genus. *P. ostreatus* ranks second only to *Agaricus bisporus* (button mushroom) in global fruiting body production, with 875,000 tons reported in 1997, representing the most recent official statistics available (Gry and Andersson 2014; OECD 2015). It is native to all continents of the northern hemisphere, with cultivation tracing back to the 1900s in the United States (OECD 2015). Now, it is cultivated worldwide on various substrates such as banana fronds, cereal straws, coffee grounds, cotton, leaves, and stalks (OECD 2015).

### 3.1 Risk Assessment

*Pleurotus* spp. are generally regarded as safe for consumption. Like basidiomycetes in general, *Pleurotus* spp. mycelia do not produce any ascomycete toxins. van Dam et al. (2024) found biosynthetic gene clusters (BGCs) related to Ochratoxin A in *P. ostreatus*, but the core genes required to synthesize it were absent. Published reports of allergic reactions are extremely limited, and the risk of autoimmune reactions and proteinaceous toxins is diminished by heating (van Dam et al. 2024). Any possible toxin presence would be likely at nontoxic concentrations with higher levels in the fruiting body rather than in mycelium.

Al‐Deen et al. (1987) and Juntes et al. (2009) report that although no poisonings have been reported for cooked *P. ostreatus*, there are reports that consuming large amounts of raw *P. ostreatus* fruiting bodies could result in adverse effects (Al‐Deen et al. 1987). The primary cause of concern in raw *P. ostreatus* is attributed to proteinaceous and water‐soluble toxic compounds, which are covered later in this review.

*P. ostreatus* safety concerns are sometimes attributed to heavy metal accumulation (e.g., Berger et al. 2022), but that topic is not covered in detail in this review. Muszyńska et al. (2015) demonstrated that the mycelium accumulates lower concentrations of heavy metals than fruiting bodies. Lectins are not within the scope of this paper, but it is worthwhile to mention that *Pleurotus* lectins are an example of defense‐related compounds primarily associated with the fruiting body, and are only temporarily present in vegetative mycelium before fruiting (Nikitina et al. 2017).

Baars et al. (2004) reported on respiratory problems encountered by workers associated with *P. ostreatus* fruiting body cultivation. This adverse effect is related to spore production by the *P. ostreatus* fruiting body and does not affect the mycelium.

## 4 Main Mycotoxins

Mycotoxins are defined by Alshannaq and Yu (2017) as toxic secondary metabolites synthesized by filamentous fungi, capable of inducing acute or chronic toxicity in animals and humans. These compounds are generally very stable; thus, they may accumulate over time in food and feed, posing a significant health risk. The variation in basidiomycete mycotoxin concentrations between species is not only an evolutionary adaptation to deter predators. Factors such as nutrient access, altitude, light exposure, and developmental stage also affect mycotoxin production (Enjalbert et al. 1989; Qi et al. 2020). Certain species may have evolved an overproduction of a toxin normally present in smaller concentrations as a primary metabolite (Faulstich and Cochet‐Meilhac 1976).

**FIGURE 1:**
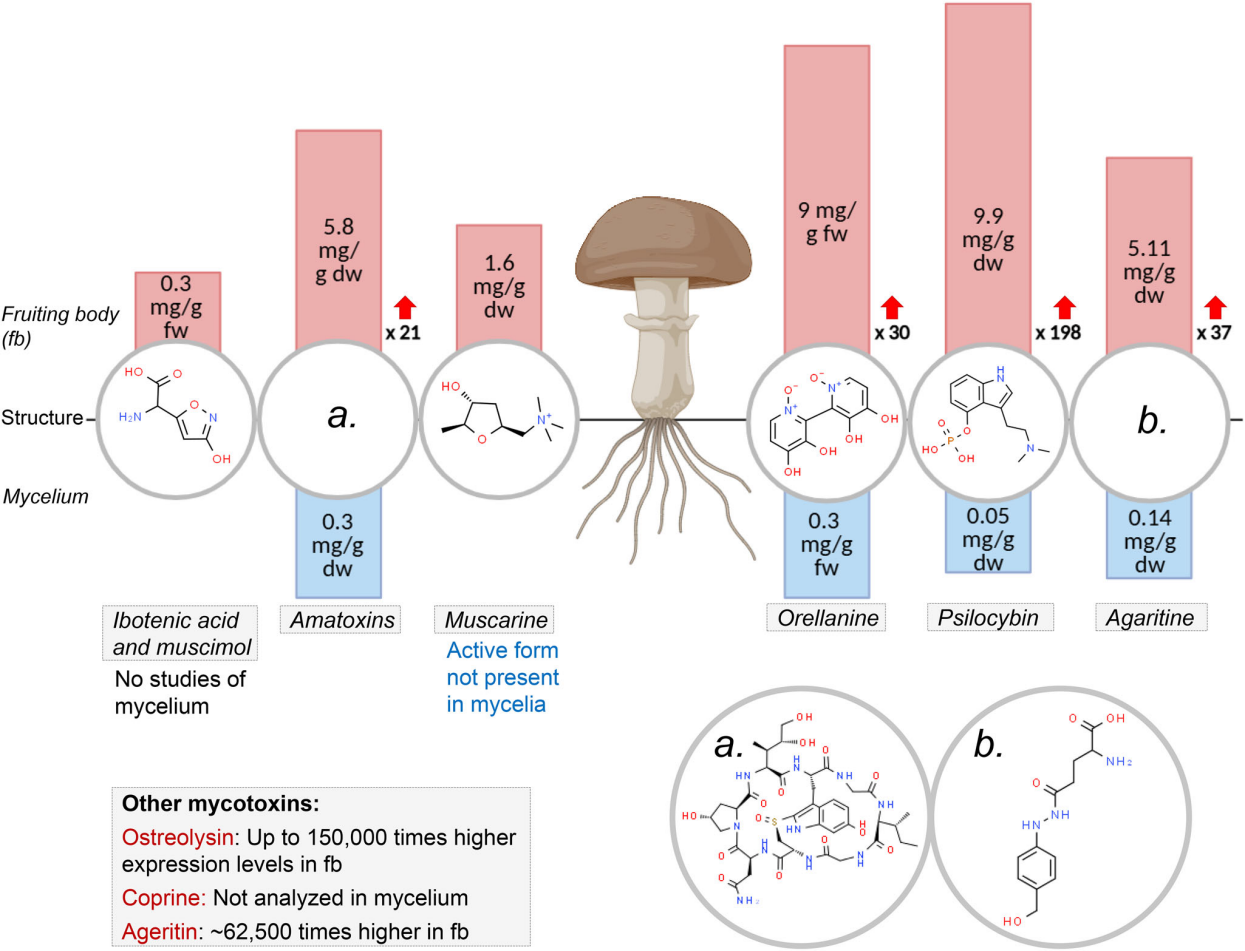
Concentration differences of various mycotoxins between the fruiting body and mycelium. dw = dry weight; fb = fruiting body; fw = fresh weight. The fruiting body in the figure is a generic representation, not based on any specific fungus. Staple sizes are meant to be indicative, not to scale. Graphic made at Biorender. Chemical structure graphics retrieved with permission from Chemspider.

In basidiomycetes, mycotoxin production is often localized to specific structures within the organism; for example, agaritine and amatoxins are predominantly found in the fruiting body, particularly in the cap (Berne et al. 2007; Kaya et al. 2015). The fruiting body usually grows exposed outside the medium, which subjects it to different stress factors than the mostly underground vegetative mycelium. Some basidiomycete mycotoxins are toxic to fungivores, which could explain why expression is mainly localized to the fruiting body, with lower concentrations in the mycelium. This review found no toxins that are unique to mycelium. Some toxins detected in mycelium, for example, OlyA/PlyB, were only temporarily present, indicating that toxin synthesis can also be dependent on the development stage. The underlying expression mechanisms are complex, but this section provides an overview of the known factors that influence the production of the most significant basidiomycete mycotoxins, with a particular focus on the genus *Pleurotus*. The main findings have been compiled into Figure 1.

More than 300 mycotoxins have been identified, with the majority produced by ascomycetes, including the genera *Aspergillus*, *Penicillium*, *Fusarium*, *Alternaria*, and *Claviceps* that account for most of the mycotoxin contamination of food (Alshannaq and Yu 2017). While the EU legislation sets maximum levels for certain ascomycete toxins in Commission Regulation (EC) No 1881/2006 (European Union, n.d.‐b), there is no clear guidance on main basidiomycete mycotoxins. However, EFSA has assembled a list of certain toxicity‐related keywords for assessing novel food literature search titled *Systematic literature search to assist EFSA in the preparatory work for the safety assessment of Novel Food applications and Traditional Food notifications* (EFSA 2020b). All basidiomycete mycotoxins in the list have been included in Table 1, along with information on known occurrences of the toxins throughout basidiomycetes and in the genus *Pleurotus*. Literature sources are included in the table, together with references to later sections with more detailed explanations.

**TABLE 1:** List of all basidiomycete toxins included in the EFSA (2020b) toxicity‐related keyword list.

Toxin name, synonyms separated by semicolons	Occurrence in Basidiomycota	Occurrence in the genus *Pleurotus*
2‐Amino‐5‐hexynoic acid	*Trogia venenata* (Zhou et al., 2012)	Not determined (ND)
2S‐2‐Amino‐5‐chloro‐4‐hydroxy‐5‐hexenoic acid	*Amanita* genus, *Cortinarius claricolor* (Hatanaka et al., 1994)	ND
Acromelic acid	*Clitocybe acromelalga* (Fushiya et al., 1990)	ND
Agaricin; agaric acid; agaricic acid; alpha‐cetyl citric acid	Polypores, i.e., genera *Fomitopsis, Laricifomes*, *Polyporus*, *Ischnoderma* (MedChemExpress, n.d., PubChem, n.d.‐a)	ND
Agaritine	Widely present in many fungi (see Section 4.2)	Possible false positive in one study (see Section 4.2)
Ageritin; edulitin; gambositin; ostreatin	See Section 4.9	Ostreatin is present; little to no data on toxicity (see Section 4.9)
Amanitin; amatoxin	Harmful levels in *Amanita*, *Lepiota*, and *Galerina*. Nontoxic levels detected in several widely consumed fungi from the order Agaricales (see Section 4.3)	Likely at nontoxic levels. Present in many widely consumed fungi of the order Agaricales (see Section 4.3)
Bufotenin	Genus *Amanita* (Alonzo & Fu, 2023)	ND
Clitidine; chrytidine	*Clitocybe acromelalga* (syn. *Paralepistopsis*) (Wurita et al., 2019)	ND
Coprine	Principal toxin in, but not exclusive to, *Coprinopsis* genera (see Section 4.4)	Claimed detected but not quantified (see Section 4.4)
Cucurbitacin	Present in the genera *Russula, Hebeloma, Leucopaxillus* (Clericuzio et al., 2007, 2012)	Possible. One study claims presence in *P. sajor caju* (Devi and Krishnakumari, 2015)
Cycloprop‐2‐ene carboxylic acid	*Russula subnigrans* (Matsuura et al., 2009)	ND
Fasciculol steroid	No records outside of genus *Hypholoma* (Chuluunbaatar et al., 2019)	ND
Gymnopilin	No records outside genus *Gymnopilus* (PubChem, n.d.‐b)	ND
Hebevinoside	No records outside *Hebeloma vinosophyllum* (Fujimoto et al., 1991)	ND
Ibotenic acid	Only in a few *Amanita* species (see Section 4.5)	ND (see Section 4.5)
Illudins; lampterol	No records outside of the genera *Craterellus*, *Clitocybe*, *Coprinopsis*, *Omphalotus* (see Section 4.6)	Not detected (Kanamori‐Kataoka et al., 2006)
Muscarine	Widely distributed in the order Agaricales. Principal toxin in e.g., genera *Clitocybe* and *Inocybe*, present in nontoxic levels in widely consumed fungi from other orders, e.g., *Boletus* (order Boletales) and *Lactarius* (order Russulales) (see Section 4.7)	Not detected, but as members of the order Agaricales, *Pleurotus* spp. could theoretically contain muscarine at nontoxic levels (see Section 4.7)
Muscimol	Only in a few *Amanita* species (see Section 4.5)	ND (see Section 4.5)
Orellanin(e); cortinarin	Unique to *Cortinariaceae* (see Section 4.8)	Unlikely. Presence falsely claimed in *P. ostreatus* in one review article (see Section 4.8)
Ostreolysin	Unique to genus *Pleurotus* (see Section 4.1.1)	Yes, associated with fruiting body development (see Section 4.1.1)
Phallotoxins	Reported to be only produced by the genera *Amanita*, *Galerina*, *Lepiota*, and *Pholiotina* fungi (see Section 4.3)	ND (see Section 4.3)
Polyporic acid	Polypores, e.g., *Hapalopilus nidulans*	ND
Psilocin; psilocybin	Only in a few mycorrhizal fungi (see Section 4.10)	Unlikely*. Pleurotus* spp. are not mycorrhizal (see Section 4.10)
Saponaceolide	*Tricholoma terreum* and *T. saponaceum* (PubChem, n.d.‐c)	ND
Tricholomic acid	*Tricholoma muscarium* (Takemoto & Nakajima, 1964)	ND
Ustalic acid	*Tricholoma ustale* (PubChem, n.d.‐d)	ND
Virotoxins	Present in amatoxin‐ and phallotoxin‐producing species (see Section 4.3)	ND (see Section 4.3)

Narrowing mycotoxins to taxonomic ranks is not viable. A single species can synthesize multiple mycotoxins, and the same mycotoxins can be produced in multiple taxonomically distant genera. Moreover, numerous deadly mycotoxins, for example, amatoxins, are suggested to be present in widely consumed species, but at nontoxic concentrations (Faulstich and Cochet‐Meilhac 1976).

This section gathers information on the taxonomic and structural distribution of the most relevant basidiomycete mycotoxins. Key findings are summarized in Tables 1 and 2. Where data are available, toxin concentrations are reported in Table 2. However, a limitation that applies both to this section and to the literature reviewed is that not all cited studies report toxin concentrations relative to dry weight, nor do they give the moisture content from which dry weight could be calculated. This lack of standardized reporting limits comparability, as moisture content can vary significantly depending on fungal structure and developmental stage. Nevertheless, studies reporting toxin concentrations on a dry‐weight basis were prioritized over those using wet weight, and the basis of measurement is indicated for all concentrations reported in this paper.

**TABLE 2:** Distribution of mycotoxins in structures of basidiomycetes and *Pleurotus* spp. where applicable.

Name	Related compounds	Concentration in structures of basidiomycetes and genus *Pleurotus*	Reference
Agaritine	Inadequate toxicity data	*A. bisporus* fruiting body: 0.170–0.511 g/100 g dw *A. bisporus* mycelium: 0.014–0.032 g/100 g dw	Speroni et al., 1983
Amatoxins	α‐Amanitin, β‐amanitin, γ‐amanitin, ε‐amanitin, amanullin, amanullic acid, amaninamide, amanin, and proamanullin	*A. phalloides* fruiting body 2.8 mg α‐amanitin/g dry weight (dw) *A. phalloides* mycelium 0.024 mg α‐amanitin/g dw	Kaya et al. (2015)
Coprine	Toxic degradation product cyclopropenone	No data on presence in mycelium	—
Ibotenic acid	Muscimol, ibotenic acid is the inactive form	Concentrations decrease in parts of the fruiting body closer to the ground. No data on presence in mycelium	Tsunoda et al. (1993a)
Illudins	Large family of sesquiterpene compounds (see Section 4.6)	Present both in fruiting bodies and mycelium. No data within a single species	See Section 4.6
Muscarine	Similar structure to acetylcholine, mimics function	Only known to be expressed in fruiting bodies of *Clitocybe* and *Inocybe* species. Only nontoxic, phosphorylated form detected in mycelium	Dörner et al. (2024)
Muscimol	Similar structure to GABA, mimics function	Concentrations decrease in parts of the fruiting body closer to the ground. No data on presence in mycelium	Tsunoda et al. (1993a)
OlyA/PlyB (ostreolysin)	Pleurotolysin	Present in *P. ostreatus* fruiting body, absent in *P. ostreatus* mycelium	Berne et al. (2007)
Orellanine	Orellinin and orellin. Additionally, fungi modify orellanine into cortinarin A, B, and C	*C. orellanus* fruiting body (cap) 9.4 mg/g dw. *C. orellanus* fruiting body (stem) 4.8 mg/g dw. *C. orellanus* mycelium 0.3 mg/g dw	Koller et al. (2002)
Ostreatin	Ageritin, edulitin, eryngitin, gambositin	Ostreatin and related ageritin present in lower concentrations in mycelium compared to fruiting body	Ragucci et al. (2021); van Dam et al. (2024)
Phallotoxins	Phalloidin, prophalloin, phalloin, phallisin, phallacidin, phallacin, and phallisacin	Follow a similar distribution and concentration as amatoxins	Kaya et al. (2015)
Psilocybin	Psilocybin toxicologically inactive but converted into toxic psilocin in the human body. Other related compounds are aeruginascin, baeocystin, norbaeocystin closely related to psilocybin	*Psilocybe* spp. fruiting body 9.9 mg/g dw *Psilocybe* spp. mycelia 0.046 mg/g dw	Waldbillig et al. (2023)
Virotoxins	Viroidin, alaviroidin, deoxoviroidin, deoxoviroisin, and viroisin	Not determined (ND)	—

Furthermore, short overviews of advances in detection are included for each toxin. However, detailed discussion is limited by the small and highly heterogeneous body of available studies. Due to differences in approaches, matrices, and reporting, clear trends cannot yet be reliably identified.

### 4.1 Aegerolysins

Multiple sources agree that the principal toxic compounds behind intoxication from ingesting raw *P. ostreatus* are cytolytic water‐soluble proteins, most likely the aegerolysins OlyA/PlyB (formerly known as ostreolysin) and pleurotolysin (Al‐Deen et al. 1987; Gry and Andersson 2014; Juntes et al. 2009; Lima et al. 2012). Berne et al. (2007) note that as aegerolysins are pH‐inactivated, they are only toxic to humans if administered intravenously. Still, intoxication symptoms from aegerolysins have been reported following raw *P. ostreatus* ingestion, likely due to the large quantities of fruiting bodies consumed at once (Al‐Deen et al. 1987). *P. ostreatus* mycelium contains significantly lower levels of aegerolysins than the fruiting body, and their expression is only initiated at the beginning of fruiting, starting from mycelium aggregation and hyphal knots (Kraševec and Skočaj 2022).

After analyzing the genomes of over 38 *Pleurotus* strains, van Dam et al. (2024) discovered that many different aegerolysins are expressed throughout the genus. Additionally, expression levels were reported to be up to 150,000 times lower in the mycelium than in the fruiting body.

Recent advances in the detection and characterization of aegerolysins have been largely driven by research in pest control and biomedicine. In particular, cryo‐electron microscopy has contributed to describing the toxicological mechanism of aegerolysins, while bioassays by Panevska et al. (2021) have demonstrated their specific toxicity against certain insects (Ota et al. 2013).

Al‐Deen et al. (1987) studied the toxicological properties of a *P. ostreatus* fruiting body extract made by evaporating water from an 8 g fresh sample until 1 g was left. The authors did not report the initial or final moisture content. The study described symptoms from repeated oral administration of the extract. The LD50 value for mice was determined as 319 mg extract/kg bodyweight (bw)/day, which is equivalent to a 70 kg person consuming 2.8 kg of fresh *P. ostreatus* per day, which is not realistic. Moreover, the obtained LD50 value may have been influenced by the presence of other toxic compounds, as no purification or identification steps were performed for the aegerolysins described in the study. This work is revisited in Section 4.9 in light of more recent studies. However, other studies have also demonstrated inflammation and microabscesses in the liver and hemorrhages in the intestine, heart, and lungs caused by *P. ostreatus* consumption (Juntes et al. 2009).

#### 4.1.1 OlyA/PlyB (Ostreolysin)

OlyA/PlyB form a complex, formerly known as ostreolysin, which has been characterized by Berne et al. (2007) as a heat‐labile, acidic, and proteinaceous cytolysin. The LD50 of OlyA/PlyB for mice has been reported as 1.17 mg/kg bodyweight, intravenously administered (Juntes et al. 2009). OlyA/PlyB is produced by symbiotic microbes and encoded by select gene sequences in *P. ostreatus*. While it is readily present in the fruiting body, Berne et al. (2007) assess that it is absent in *P. ostreatus* mycelium.

Berne et al. (2007) argue that the expression of OlyA/PlyB is related to its role as a growth promoter, initiating the formation of primordia and developing them into a fruiting body. According to them, fruiting initiation by OlyA/PlyB is simultaneous with the inhibition of mycelial growth.

The origin of OlyA/PlyB is argued to be in bacterial sporulation mechanisms (Berne et al. 2007; Cho et al. 2003). Hemolytic *P. ostreatus* aegerolysins structurally similar to *Clostridium bifermentus* proteins have been discovered by Frangež et al. (2017). Furthermore, several hemolytic *Pseudomonas* (e.g., *Pseudomonas putida*) are known to live on *P. ostreatus* cultivated on composted material (Cho et al. 2003). Cho et al. go as far as to emphasize the role of adjunct *Pseudomonas* by correlating poor fruiting body harvest with non‐composted growth medium lacking OlyA/PlyB‐producing species. However, fruiting is not entirely dependent on promoter flora, as aegerolysin protein‐encoding genes have been found in *P. ostreatus* by S.‐H. Lee et al. (2009). The biological role of ostreolysin is, in addition to fruiting promotion, attributed to defense against molds, thus the cytolytic property (Sepcić et al. 2004). OlyA/PlyB is reported to be absent in *P. ostreatus* vegetative mycelium, only starting to appear when primordia begin to develop (Berne et al. 2007).

#### 4.1.2 Pleurotolysin

Pleurotolysin was isolated by Tomita et al. (2004) from *P. ostreatus* and identified as a two‐component cytolysin consisting of pleurolysin A and pleurolysin B. The cytolytic property is proposed to result from a combined sphingomyelin‐specific effect of both components (Tomita et al. 2004). The disrupting effect on erythrocytes indicates hemolytic activity. Bernheimer and Avigad (1979) described a similar hemolysin, most likely referring to pleurotolysin. The authors claim that pleurotolysin is denatured by heat, thus inhibiting the hemolytic properties. Pleurotolysin is present in eukaryotes, prokaryotes, and archaea with roles in programmed cell death (Frangež et al. 2017). It is weakly expressed in aggregating *P. ostreatus* mycelium in hyphal knots, but at fruiting, its expression increases fourfold in primordia‐associated mycelium, and 90‐fold in the fruiting body basidiocarp, suggesting a role in fruiting initiation (Kraševec and Skočaj 2022).

### 4.2 Agaritine

Agaritine is a potentially carcinogenic nonprotein amino acid and a derivative of phenylhydrazine, which occurs in many widely consumed fungi, for example, *Agaricus*, *Leucoagaricus*, and *Macrolepiota*. *P. ostreatus* has been suggested to produce agaritine, but has tested negative in multiple screenings (Stijve and Pittet 2000). The report that initially caused concern is suspected of providing a false positive agaritine presence in *P. ostreatus* (Stijve and Pittet 2000). Furthermore, agaritine is readily destroyed upon cooking and freezing, which may reduce its toxicity in foods (Schulzová et al. 2002). The instability of agaritine also complicates the achievement of high analytical precision.

Agaritine research has been most active in the early 21st century with limited recent developments. The analytical methods used have been mostly liquid chromatography‐mass spectrometry (LC‐MS) and fluorescence derivatization (Kondo et al. 2006; Nagaokaa et al. 2006). Electrospray ionization tandem mass spectrometry has achieved limits of quantification as low as 0.01 µg/g (Kondo et al. 2006).

In *A. bisporus*, agaritine is suggested to be a fungicide that improves competitiveness against molds (Speroni et al. 1983). It is distributed throughout the fruiting body (1.70–5.11 mg/g dry weight), with the highest concentrations in the cap and gills, lower in the stem, and in significantly smaller concentrations in mycelium (0.14–0.32 mg/g dry weight) (Ross et al. 1982; Speroni et al. 1983). Furthermore, Speroni et al. (1983) found agaritine concentrations in the fruiting body to increase sevenfold during fruiting. They also reported a significantly higher level of agaritine (0.73–7.39 mg/g dry weight) in fruiting bodies grown on a synthetic compost consisting of hay, pelleted corn cob, potash, and gypsum, than in fungi grown on conventional compost material (0.47–3.55 mg/g dry weight) dominant in horse manure, but also containing brewer's grain, poultry manure, and gypsum.

### 4.3 Amatoxins, Phallotoxins, and Virotoxins

Amatoxins, phallotoxins, and virotoxins are chemically related, but toxicologically different, cyclic oligopeptides. Amatoxins have been detected in low concentrations in nontoxic basidiomycetes; still, evidence largely suggests that only a few genera have evolved to produce them in harmful levels (Faulstich and Cochet‐Meilhac 1976; Walton et al. 2010). Walton et al. (2010) identified nontoxic species from genera unrelated to the known toxic amatoxin producers. Moreover, since Faulstich and Cochet‐Meilhac (1976) documented the nontoxic presence of amatoxins in widely consumed fungi, it is possible that *Pleurotus* species contain negligible amounts of amatoxins and phallotoxins.

Radioimmunoassays, such as those employed by Faulstich and Cochet‐Meilhac (1976), were among the earliest methods developed for amatoxin analysis. However, the majority (70%) of studies conducted today rely on chromatographic methods for analysis, allowing simultaneous amatoxin and phallotoxin analysis (Barbosa et al. 2023). Current trends in amatoxin detection increasingly emphasize rapid and simplified methods suitable for clinical diagnostics and field testing (Barbosa et al. 2023). On the other hand, LC‐MS methods like quadrupole tandem and orbitrap MS have achieved unparalleled sensitivity and reproducibility. This is likely to enable the detection and quantification of amatoxins at concentrations as low as 0.5 ng/mL in injection samples (Yang et al. 2024).

There are consistent and repeating indications of uneven distribution of amatoxins and phallotoxins between fungal structures. Kaya et al. (2015) demonstrated that the fruiting body had 21 times higher concentrations of amatoxins than the mycelium, with a similar distribution of phallotoxins. *Galerina marginata* (funeral bell) fruiting body contained twice the amatoxin concentration as the respective mycelium (Akata et al. 2020; Sgambelluri et al. 2014). However, the fruiting body and mycelium were from two separate studies. Additionally, Kaya et al. (2015) are among several researchers who have observed increasing amatoxin concentrations in fungal structures located higher above the ground, something that has been observed with other species and mycotoxins as well.

Amatoxins are a group of thermostable octapeptides with nine identified compounds (Kaya et al. 2015). Throughout the literature on amatoxins, α‐amanitin has consistently been the most prevalent (e.g., Akata et al. 2020; Hu et al. 2012; Kaya et al. 2015). The toxic distribution of hepatotoxic amatoxins is based on absorption in the gastrointestinal tract, from where they are distributed to the liver. They are named after the genus *Amanita*, although not all *Amanita* species synthesize these toxins. Moreover, amatoxins are not unique to *Amanita*, and literature suggests they are also produced at harmful levels by the genera *Lepiota* and *Galerina*, and one species belonging to the genus *Pholiotina* (Diaz 2018). Diaz conducted an extensive review of amatoxin poisonings, with the conclusion that no poisonings have been reported outside these genera. However, the widely consumed fungi *Agaricus silvaticus* (scaly wood mushroom), *Boletus edulis* (cep, porcini), and *Cantharellus cibarius* (golden chantarelle) have been found to contain detectable, but non‐harmful, levels of amatoxins (Faulstich and Cochet‐Meilhac 1976). The authors speculated that the role of amatoxins is in regulating protein synthesis, possibly indicating a nontoxic presence in most basidiomycetes at a baseline concentration in the range of approximately 1–10 ng/g wet weight. This observation suggests that while amatoxins are considered secondary metabolites when found at toxic levels, they may actually be primary metabolites that certain species have evolved to overproduce.

Kaya et al. (2015) measured low total amatoxin concentrations of 0.27 mg/g dry weight in *A. phalloides* (death cap) mycelium, while an amount of 5.78 mg/g dry weight was detected in the fruiting body measured as a whole. A similar distribution of phallotoxins was also detected. Furthermore, Kaya et al. (2013) investigated variations in amatoxin concentrations across different structures of *A. phalloides* var. *alba* fruiting body. Their findings showed that amatoxin levels increased in structures higher above the ground. The highest total amatoxin concentrations were detected in the cap, 4.42 mg/g dry weight, followed by the stem (2.73 mg/g dry weight), while the volva contained the lowest levels (0.99 mg/g dry weight). Stötefeld et al. (2012) explained this concentration increase as possibly reflecting protection against arthropods. However, beyond this, the biological roles of amatoxins have not been studied.

Phallotoxins are a class of hepatotoxic bicyclic heptapeptides (seven amino acids), with seven identified compounds (Sgambelluri et al. 2014). The name phallotoxin is derived from *A. phalloides*, the fungus that is responsible for most of the fatal mushroom poisonings (Diaz 2018). Phallotoxins are reported to be produced in toxic concentrations only by the genera *Amanita*, *Galerina*, *Lepiota*, and *Pholiotina*, species that also produce amatoxins (Walton et al. 2010). Phallotoxins are heat‐sensitive and are not absorbed in the gastrointestinal tract; thus, they are not considered responsible for *Amanita*, *Galerina*, *Lepiota*, and *Pholiotina* poisonings (Morel et al. 2016).

Virotoxins are a group of cyclic heptapeptides with seven identified compounds (Morel et al. 2016). They are a less studied group of cyclic peptide toxins with an LD50 value of 1.0–5.1 mg/kg for mice (Loranger et al. 1985). The name is derived from *Amanita virosa* (destroying angel). Due to their poor oral absorption in humans, they have very little clinical importance (Morel et al. 2016).

### 4.4 Coprine

Coprine is a nonprotein amino acid. It is the principal mycotoxin in toxic species of the genus *Coprinopsis*. Its toxicity is based on inhibiting the acetaldehyde dehydrogenase enzyme, leading to an alcohol flush reaction if consumed simultaneously with ethanol. It is claimed in the Human Metabolome Database (n.d.) that l‐coprine has been detected in *P. ostreatus*, *A. bisporus*, and other fungi, but not quantified. No information on coprine presence in mycelium is available. Its biological role in fungi has not been clarified.

Recent advances in coprine detection have been driven largely by the development of electrochemical sensing. The work by Tkach et al. (2024) presents a theoretical evaluation of coprine detection using a modified electrode. Previously, coprine has been mostly analyzed by chromatographic methods for both food and biomedical applications. Now, similarly to trends in amatoxin detection, these developments have been motivated by the need for fast, cost‐effective, and portable detection tools for clinical and field use.

### 4.5 Ibotenic Acid and Muscimol

Ibotenic acid (ibo) and muscimol, also known as agarin or pantherine, are the principal mycotoxins in *Amanita muscaria* (fly agaric). Both are thermostable derivatives of isoxazoles (Berger and Guss 2005). The structure of muscimol, 3‐hydroxy‐5‐amino‐methylisoxazole, resembles gamma‐aminobutyric acid (GABA), the chief mammalian inhibitory neurotransmitter, altering neuronal activity and inducing toxicity and psychoactive effects (Johnston 2014). According to Obermaier and Müller (2020), ibotenic acid structurally resembles another neurotransmitter, glutamate. Ibotenic acid and muscimol are sometimes deliberately ingested for this reason. In the United States, the FDA has ruled that they are unapproved food additives, as is *A. muscaria*, in which they are found (FDA 2024b). The LD50 for orally administered muscimol in rats is 45 mg/kg bw and 129 mg/kg bw for ibotenic acid (ChemicalBook n.d.; Theobald et al. 1968). Human deaths are rarely reported for muscimol. Opossums were proven to develop an aversion to muscimol‐containing fungi after being intoxicated, indicating a possible defense role against mammals (Camazine 1983).

Recent developments in the analysis of ibotenic acid and muscimol have primarily focused on applications in clinical and forensic toxicology, as well as food safety. During the 2020s, liquid chromatography–tandem mass spectrometry (LC‐MS/MS) and ultra‑performance liquid chromatography–tandem mass spectrometry (UPLC‐MS/MS) have significantly improved analytical sensitivity below ng/mL detection limits in biological materials (Xu et al. 2020; Zhao et al. 2022). Advances in sample preparation, including solid‐phase extraction and direct derivatization, have made methods more accessible while allowing the simultaneous detection of multiple toxins (Zhao et al. 2022). Furthermore, paper spray ionization coupled with MS/MS has been successfully applied as a rapid screening method for ibotenic acid in fungal material, achieving detection limits as low as 1.3 µg/mL (Luo et al. 2025).

Not all *Amanita* species produce ibotenic acid and muscimol (Obermaier and Müller 2020). Obermaier and Müller found a BGC responsible for the production of ibo. Genome analysis performed on non‐ibotenic acid‐producing *Amanita* species indicated the lack of the ibo BGC. Conclusively, ibotenic acid production, and thus muscimol production, is suggested to be possible only in the genus *Amanita* subgenus *Amanita*, that is, species *Amanita crenulata* (has the ibo BGC but it is not expressed), *A*. *muscaria*, and *Amanita pantherina*.

However, Berger and Guss (2005) include other species of *Amanita* (*Amanita cokeri*, *Amanita solitaria*, *Amanita strobiliformis*, *Amanita cothurnata*, *Amanita gemmata*, *Amanita citrina*) and *Panaeolus campanulatus* as isoxazole‐synthesizing fungi. Nonetheless, the sources cited are mainly unfounded field books and guides. Furthermore, a literature search performed with the search engines Google, Google Scholar, jurn.org, and the University of Helsinki databases, by using keywords “Ibotenic acid” or “Isoxazole” and “*Panaeolus*” or “*Amanita*,” does not provide any studies confirming Berger's and Guss’ (2005) claim.

Ibotenic acid and muscimol concentrations decrease in parts of the fruiting body closer to the ground (Tsunoda et al. 1993a). However, it cannot be determined from this whether they are present in even lower concentrations or absent in mycelium. In *A. muscaria*, ibotenic acid and muscimol concentrations are highest in the cap, with the stem containing about half of that (Tsunoda et al. 1993a). The toxin concentrations in the fruiting body are not reported to correlate with the growth location or the fruiting body size; instead, the concentration differences are attributed to individual variation in toxin expression (Tsunoda et al. 1993b). No data can be found on the presence in mycelium.

### 4.6 Illudins

Illudins are a group of toxic, tricyclic sesquiterpenes produced by fungi, with the most well‐known ones being illudin A, B, M, and S. These compounds are highly toxic due to their genotoxic and cytotoxic properties (Casimir et al. 2023). In mouse models, the LD50 value of illudin S has been reported as 5 mg/kg bw (Kanamori‐Kataoka et al. 2006). Because of their selective cytotoxicity, illudins have attracted interest as potential antitumor and antibacterial agents. Other related compounds in the sesquiterpene illudin family are, for example, illudalic acid, illudacetalic acid, illudinine, illudinic acid, and illudosin (Anchel et al. 1950; Woodward and Hoye 1977).

Detection limits as low as 36 pg/µL, with good recovery and linearity across the picogram‐to‐nanogram range, were already reached in the 2000s using gas chromatography‐mass spectrometry (Kanamori‐Kataoka et al. 2006). Given the availability of multiple highly sensitive analytical techniques for illudins today, like desorption electrospray ionization–tandem mass spectrometry (DESI‐MS/MS), which additionally enables spatially resolved analysis (imaging) of samples, recent research has increasingly turned toward toxicological studies (Matsui et al. 2024). These include studies examining interactions with biomolecules, such as DNA (Casimir et al. 2023).

Historically, illudins have been extensively investigated for their anticancer potential. Kelner et al. (1990) demonstrated that illudins exhibit selective toxicity toward various tumor cell lines, including breast carcinoma, epidermoid carcinoma, leukemia, lung, and ovarian cells from different species, including humans. The semisynthetic derivative irofulven, developed from illudin S, is currently undergoing phase II clinical trials in the United States as an FDA‐approved Investigational New Drug (Chaverra Muñoz et al. 2022). However, the precise antitumor mechanism of illudins remains under investigation.

The name “illudins” derives from *Omphalotus illudens*, the species from which illudin S and M were first isolated (Anchel et al. 1950). Illudins A, B, G, H, and S have only been reported from *Omphalotus* species, in the order Agaricales (Gonzalez Del Val et al. 2003). Outside of this genus, illudin M has been detected in *Clitocybe phosphorea*. However, Gonzalez Del Val et al. (2003) suggested that this species may actually belong to *Omphalotus*, as both genera have previously been synonymized due to their shared bioluminescent properties (Cardillo et al. 1992). However, differences in the biosynthetic pathways of illudins have been observed between species of the genera *Clitocybe* and *Omphalotus* (Cardillo et al. 1992).

Gonzalez Del Val et al. (2003) also identified three novel illudins, Illudin I, I2, and J2, and illudinic acid in 10 *Coprinopsis* species (order Agaricales). The *Coprinopsis* species listed were, for example, *Coprinopsis atramentarius*, *Coprinopsis cinerea*, *Coprinopsis friesii*, and *Coprinopsis radiata*. As discussed in Section 4.4, *Coprinopsis* is also the only known genus that produces the structurally unrelated compound coprine. Notably, *C. atramentarius* is the only species known to produce illudin C, C2, and C3 (I. K. Lee et al. 1996). In addition, non‐illudin sesquiterpenes, such as bovistol B and D, and strossmayerin, have been detected in *Coprinopsis* species (Banks et al. 2020). Illudins have also been detected in the order Cantharellales, where *Craterellus cornucopoides* was found to contain the illudins F and T among other sesquiterpenes (Guo et al. 2017).

Kanamori‐Kataoka et al. (2006) investigated the presence of illudin S in *P. ostreatus*, *L. edodes*, and *Panellus serotinus*, species that have been implicated in poisoning cases based on misidentification due to visual likenesses with the poisonous *Ophiopogon japonicus*. However, no illudin was detected in them. By contrast, illudin S was detected in *O. japonicus*.

Illudinic acid, a sesquiterpene structurally related to illudin C, has demonstrated antibacterial activity against *Staphylococcus aureus* (Dufresne et al. 1997). The producing fungus was identified as a basidiomycete, although no further taxonomic classification was determined. Similarly, illudins isolated from *Coprinopsis episcopalis* mycelium demonstrated antibacterial properties against methicillin‐resistant *S. aureus* (Gonzalez Del Val et al. 2003).

Illudin production has been more extensively studied in fungal mycelia than in fruiting bodies, limiting the ability to compare concentration distributions between these structures. Since various species can produce illudins in mycelial culture, multiple methods have been developed to ensure a continuous supply of these compounds. Thus, most available data reflect optimized laboratory yields rather than natural concentrations. For instance, with fermentation process optimization, substantial illudin S concentrations of 4 g/L in *O. illudens* culture medium were reached by McMorris et al. (2000). Similarly, Chaverra Muñoz et al. (2022) increased illudin M production in *Omphalotus nidiformis* mycelial cultures from 0.038 to 0.94 g/L by addressing biosynthetic bottlenecks. Cardillo et al. (1992) also isolated 100 mg of illudin M and 150 mg of illudinine from 1.5 g of dehydrated *C. phosphorea* mycelium, which is equal to 1 g illudin M and 1.5 g illudinine per 1 L culture liquid. No fruiting body reference values were provided in the study. Guo et al. (2017) reported the presence of illudins F, M, and T in *C. cornucopoides* mycelial cultures but did not quantify their concentrations. Thus, it can be concluded that many factors affect illudin concentration variation.

Illudins are not restricted to mycelium. In *O. illudens* fruiting bodies, illudin S concentrations of 23 µg/g wet weight have been measured, while in *O. japonicus* concentrations ranged from 1.9 to 318 µg/g wet weight, depending on the specimen (Kanamori‐Kataoka et al. 2006).

Due to methodological differences among studies, direct comparison of illudin concentrations between mycelium and fruiting bodies is not applicable. Current evidence suggests that illudins occur only in species of the order Agaricales, predominantly in the genera *Omphalotus* and *Coprinopsis*, but also in *Craterellus* and *Clitocybe*. Illudins are present in both mycelial and fruiting body tissues with highly variable concentrations. The widely consumed Agaricales species *P. ostreatus* and *L. edodes* show no detectable levels of illudins (Kanamori‐Kataoka et al. 2006).

### 4.7 Muscarine

Muscarine is a tetrahydrofuran containing a quaternary ammonium. It is structurally related to acetylcholine and binds to its namesake receptor, the muscarinic acetylcholine receptor. Acetylcholinesterase cannot degrade muscarine, leading to poisoning in humans. The toxin is named after *A. muscaria*, which, despite the association, does not contain toxic amounts of muscarine (Lurie et al. 2009). Harmful concentrations of muscarine are mainly associated with the order Agaricales in genera such as *Clitocybe* and *Inocybe*, for example, in the species *Clitocybe phyllophila*, *Clitocybe dealbata*, *Inocybe rimosa*, and *Inocybe geophylla*, but also in the genera *Entoloma*, *Mycena*, and *Omphalotus* (Lurie et al. 2009). Nontoxic concentrations of muscarine have been detected in many genera outside the order Agaricales, for example, *Boletus* (order Boletales), and *Lactarius* and *Russula* (order Russulales) (Dabrowski and Sikorski 2004). It is suggested that muscarine production has evolved independently in many families of fungi. The exact biological role of muscarine is unclear. Some invertebrates are known to be susceptible to muscarine intoxication, suggesting a role in defense against insects (Tissenbaum et al. 2000).

Mass spectrometry‐based methods are the most widely applied approaches for muscarine detection, with reported detection limits as low as 0.1 µg/L (Flament et al. 2024). Recent developments have focused on rapid analytical methods suitable for clinical settings, particularly for mushroom poisonings. For example, an electrochemical sensor based on gold‐modified screen‐printed carbon electrodes has been developed for muscarine detection (Thunkhamrak et al. 2025). This platform achieved a detection limit of 4.07 µg/mL and provided results within 2 min.

Dörner et al. (2024) detected toxic concentrations of muscarine in fruiting bodies of species from the genera *Clitocybe*, *Inocybe*, and *Pseudosperma*, but did not detect it in their respective mycelia, where instead a nontoxic phosphorylated form of muscarine, 4′‐phosphomuscarine, was discovered. The concentration of this compound was found to increase in damaged mycelium. The authors speculate that 4′‐phosphomuscarine is a storage molecule that can be rapidly supplied to the fruiting body and converted to muscarine when needed.

No studies have been published on muscarine presence in *Pleurotus* spp., but as members of the order Agaricales, they could in principle contain nontoxic concentrations of muscarine. Still, existing literature suggests that the active form of muscarine is absent in the mycelium.

### 4.8 Orellanine

Orellanine is a nephrotoxic mycotoxin. Structurally, it is a bipyridine N‐oxide (2,2‐bipyridine‐3,3‐4,4‐tetrol‐1,1‐dioxide) close to the herbicides paraquat and diquat, and a known bactericide (Koller et al. 2002). The general consensus is that orellanine is exclusively found in the subgenus *Leprocybe* within the family Cortinariaceae in seven species, for example, *Cortinarius rubellus* (deadly webcap) and *Cortinarius orellanus* (fool's webcap) (Berger and Guss 2005; Dabrowski and Sikorski 2004). The same sources claim these species contain orellanine in concentrations of 9–14 mg/g dry weight. Lethal doses of orellanine are reported for mice at 33 to 90 mg/kg bw by oral administration (Dabrowski and Sikorski 2004). Orellanine is modified into orellinin, orellin, and other nephrotoxic cyclic decapeptides, for example, cortinarin A, B, and C, that have bactericidal roles in fungi (Lyons et al. 2023).

LC‐MS/MS provides high analytical sensitivity for orellanine detection, with reported detection limits as low as 30 ng/g (Shao et al. 2016). Together with high‑performance liquid chromatography (HPLC), it represents the most widely used detection techniques for orellanine analysis (Flament et al. 2024).

Lima et al. (2012) include *P. ostreatus* as a source of orellanine in a table that lists sources of mycotoxins. There is reason to believe this information is erroneous, with a possibility that *P. ostreatus* has been incorrectly categorized, not only because of the aforementioned reasons, but because the primary source cited for orellanine, Nilsson et al. (2008), does not mention *P*. *ostreatus* whatsoever. A literature search with the search engines Google Scholar, jurn.org, and University of Helsinki databases with keywords “*Pleurotus*” and “Orellanine” yields only one study by Nieminen and Mustonen (2020) claiming that *P. ostreatus* would contain orellanine, without citing a source. The form of wording suggests that Nieminen and Mustonen might have used the faulty review of Lima et al. (2012) as a source without citing it.

Regardless, Koller et al. (2002) report that *C. rubellus* stem contains significantly higher concentrations of orellanine than the mycorrhizal roots, being another mycotoxin with significantly lower concentrations in mycelium. Orellanine is suggested to have a principal role in bacterial growth inhibition, as it is known to inhibit the growth of *Bacillus subtilis* at 25 nM (Koller et al. 2002). Koller et al. determined the concentration of orellanine in *C. orellanus* mycorrhizal mycelium at 0.3 mg/g dry weight. In the same study, orellanine concentrations in the *C. orellanus* cap were measured at 9.4 mg/g dry weight and in stems 4.8 mg/g dry weight, marking a 16‐ to 31‐fold increase compared to mycelium.

### 4.9 Ostreatin and Other Ribotoxins

Ribotoxins are a group of extracellular fungal enzymes that selectively cleave ribosomal RNA, and thereby inhibit protein synthesis (Landi et al. 2020). Ostreatin is a recently identified ribotoxin found in *P. ostreatus*, where it is thought to play a role in defense mechanisms (Landi et al. 2020). Similar ribotoxins, such as ageritin, ostreatin, edulitin, eryngitin, and gambositin, have been reported in *Pleurotus* and other fungal genera. van Dam et al. (2024) found lower ostreatin levels in mycelia compared to fruiting bodies. Although ostreatin is not mentioned in the EFSA (2020b) keyword list, the related ageritin with a similar amino acid sequence is included. According to Landi et al. (2020), ageritin (not to be confused with agaritine in Section 4.2) is the first discovered fungal ribotoxin. It was extracted from *Agrocybe aegerita* (poplar fieldcap). Ageritin is known to have insecticidal, antifungal, and antiviral abilities in addition to ribotoxicity (Ragucci et al. 2021).

Recent advancements in the research of basidiomycete ribotoxins have focused on detailed structural analysis and toxicological mechanisms. These approaches involve cation‐exchange chromatography for protein purification, mass spectrometry for identification, and functional assays to evaluate their toxic activity (Landi et al. 2022; Ragucci et al. 2021).

Ragucci et al. suspect that the ribotoxin edulitin is behind food poisonings from the ingestion of raw *B. edulis*. It is destroyed at temperatures over 90°C according to them. Gambositin is a similar ribotoxin, produced by the edible *Calocybe gambosa* (St. George's mushroom), but there is no data on its toxicity, although the fungus is frequently eaten uncooked (Ragucci et al. 2021). Eryngitins from *P. eryngii* (king oyster mushroom) are ribotoxins and potential cytotoxins, but they are quickly hydrolyzed by gastric pepsin and trypsin (Landi et al. 2022).

No LD50 value is available for ostreatin, but its half‐maximal inhibitory concentration (IC50) is reported as 234 pM in rabbit protein synthesis (reticulocyte lysate) and metalloendonuclease activity (Landi et al. 2020). The stability of ostreatin is unknown, but the other known ribotoxins are largely heat‐labile and hydrolyzed in the stomach. Mentioned earlier in this review, raw *P. ostreatus* fruiting body ingestion has been reported to cause poisonings by Al‐Deen et al. (1987). They described a water‐soluble protein in *P. ostreatus* extract that was responsible for hemorrhagic and necrotic symptoms when administered intravenously. The LD50 value of the extract was 5 g/kg for mice, each gram representing 8.1 g of fresh *P. ostreatus*. The compound in question was claimed to be destroyed by gastric acid and gastrointestinal enzymes.

It is possible that Al‐Deen et al. unknowingly described ostreatin, although they suspected aegerolysins behind the poisonings. Despite proven toxicity, the experimental setup with intravenous administration does not replicate ingestion conditions. Thus, *P. ostreatus*, in respect to ostreatin and aegerolysins, can be assumed to be safe if ingested at moderate amounts, especially if heated.

Ostreatin and ageritin are structurally analogous and have an identical catalytic triad with the same enzymatic targets (Ragucci et al. 2023). Due to the limited information available on ostreatin, but its notable functional similarity, clues about its properties could be inferred from ageritin. Ageritin is a defense‐related enzyme with antifungal, genotoxic, nematotoxic, and entomotoxic properties, with a high virulence against the mold species *Alternaria alternata*, *Trichoderma asperellum*, *Botrytis cinerea*, *Colletotrichum truncatum*, and *Penicillium digitatum* (Ragucci et al. 2021; Ruggiero et al. 2019). Ageritin exhibits similar patterns to other basidiomycete mycotoxins in terms of significantly greater accumulation of a toxin in the fruiting body compared to mycelium (Ragucci et al. 2021). In the study, the *A. aegerita* fruiting body was found to contain 250 µg ageritin/g wet weight, while the concentration in mycelium was approximately 0.4 ng/g wet weight. In mycelium, ageritin was present in the intracellular space in the hyphae, which could be related to defense against fungivorous nematodes that penetrate fungal cell walls and consume cytoplasm (Kim and Knudsen 2018). Conclusively, ribotoxins are also likely to be present at significantly lower concentrations in mycelium.

### 4.10 Psilocybin and Other Tryptamine Derivatives

Psilocybin is a fungal mycotoxin that is the prodrug of psilocin, a psychotropic tryptamine (Gotvaldová et al. 2022). The 4‐phosphoryloxy‐N,N‐dimethyltryptamine structure of psilocin is similar to serotonin. This causes the hallucinogenic and intoxication symptoms from ingesting psilocybin (Lima et al. 2012). Aeruginascin, baeocystin, and norbaeocystin are related psychotropic tryptamines, but are not synthesized without psilocybin in a fungus (Gotvaldová et al. 2022).

Most analyses of psilocybin and its analogues have traditionally been performed using liquid chromatography‐based methods. High‑performance liquid chromatography with fluorescence detection (HPLC‑FL) has been successfully applied to achieve quantification limits as low as 4.4 ng/mg with good linearity of the calibration curve (Saito et al. 2005). More recently, LC‐MS/MS has enabled high sensitivity and specificity without the need for derivatization (Pego et al. 2025). Validation studies demonstrated minimal matrix interference, allowing detection limits as low as 0.5 ng/mL in fungal samples (Pego et al. 2025).

Emerging approaches include matrix‐assisted laser desorption/ionization (MALDI), which, when coupled with Fourier transform ion cyclotron resonance (FT‐ICR) mass spectrometric imaging (MSI), enables in situ visualization of psilocybin presence and distribution in a sample (H. Wang and Wang 2023). The rapid analysis enabled by this technique reduces the risk of psilocybin and related compounds degrading, a limitation associated with slower analytical methods. The technique is especially useful for forensic applications and drug monitoring, as well as in studies examining the localization of psilocybin and related compounds in fungal material.

Although named after the genus *Psilocybe*, psilocybin has a larger distribution outside the genus (Reynolds et al. 2018). Psilocybin synthesis involves four enzymes, PsiD, PsiH, PsiK, and PsiM, which are all encoded in BGCs. The enzyme BGCs are part of the genome in the genera *Galerina*, *Gymnopilus*, *Hypholoma*, *Panaeolus*, and *Psilocybe* (Reynolds et al. 2018). In addition, the genera *Inocybe, Panaeolina*, and *Pluteus* contain psilocybin‐producing species (Gotvaldová et al. 2022). Reynolds et al. mention that although psilocybin‐producing species are phylogenetically disjunct, the overlapping ecological niches, for example, engagement in dung and wood decay and mycorrhizal relationships, have led to horizontal gene transfer of metabolic gene clusters between the species.

*Pleurotus* spp. are saprobic, not mycorrhizal, and Reynolds et al. (2018) state that gene cluster transfers are more likely to happen between functionally similar species. Although not direct relatives, *A. bisporus*, another widely consumed fungus, was tested for psilocybin and psilocybin derivatives. No detectable amounts were found, indicating that not all basidiomycetes necessarily synthesize psilocybin and derivatives thereof.

Moreover, in species producing psilocybin, lower concentrations have been measured in mycelium compared to the fruiting body (Waldbillig et al. 2023). The biological roles of psilocybin and the structurally related alkaloids are unknown, except for one source mentioning slug deterrence (The University of Utah 2024). As Waldbillig et al. (2023) detected significantly higher indole alkaloid levels in the fruiting body compared to the mycelium, they even suggest *Psilocybe* spp. mycelium is a nonintoxicating material. *P. ostreatus* mycelium is proven to contain the non‐hallucinogenic indole compounds l‐tryptophan (0.019 mg/g dry weight), tryptamine (0.010 mg/g dry weight), and 5‐hydroxy‐l‐tryptophan (1.20 mg/g dry weight), which are precursors to, for example, psilocybin and serotonin (Lazur et al. 2023). However, as previously mentioned, *Pleurotus* spp. lack the four enzymes PsiD, PsiH, PsiK, and PsiM required for the biosynthetic pathway from l‐tryptophan to psilocin (Reynolds et al. 2018).

## 5 Synthesis and Limitations of the Review

Fruiting bodies are exposed to greater, although different, environmental variation and stress factors than the mycelium, which may partly explain the higher concentrations of certain mycotoxins observed in fruiting bodies. There is currently no evidence that the mycelium synthesizes mycotoxins that are not also produced in the fruiting body.

Some basidiomycete mycotoxins, such as the active form of muscarine, are known to occur exclusively in fruiting bodies. Certain basidiomycete mycotoxins have been observed in the mycelium of various species, but consistently at much lower concentrations than in the respective fruiting bodies. These include amatoxins, agaritine, ageritin, orellanine, phallotoxins, and psilocybin and related indole alkaloids. Furthermore, the concentrations of amatoxins, agaritine, ibotenic acid, muscimol, orellanine, and phallotoxins have been found to increase in parts of the fruiting body located higher above the ground. The only mycotoxins assessed in this paper for which reliable structural comparisons could not be made were the illudins. Numerous studies indicate that illudin production in mycelium can be experimentally increased to concentrations exceeding the few published values for fruiting bodies. Additionally, there are no publicly available studies reporting whether ibotenic acid, muscimol, or coprine, known to occur in the fruiting bodies of certain species, are also present in their corresponding mycelium.

The presence of OlyA/PlyB, ostreatin, and eryngitin in the genus *Pleurotus* has been confirmed by multiple independent studies. Moreover, several other basidiomycete mycotoxins have the theoretical potential to be produced by *Pleurotus* species, including coprine, amatoxins, phallotoxins, and muscarine. Given that fungal foods are typically cooked prior to consumption and pass through the acidic conditions of the stomach, the risks associated with the heat‐ and acid‐sensitive toxins, such as OlyA/PlyB, ostreatin, and eryngitin, are significantly reduced. One source claims coprine has been detected in *P. ostreatus*, but not quantified, and its presence in mycelium remains untested. Because amatoxins, phallotoxins, and muscarine have been detected throughout the order Agaricales, to which *Pleurotus* spp. belong, their presence cannot be excluded from these species. However, any such compounds are expected to occur at negligible and nontoxic concentrations in *Pleurotus* fruiting bodies, with even lower levels, if any, present in the mycelium. Furthermore, muscarine, whose presence in the fruiting body cannot be excluded, is stored in a nontoxic phosphorylated form in the mycelium.

## 6 Conclusions

All basidiomycete mycotoxins referenced in the *Systematic Literature Review for EFSA Novel Food Safety Assessment* resource (EFSA 2020b) were addressed in this paper. The findings strongly suggest that in basidiomycetes, and with no evidence of exceptions, most toxins are primarily, if not exclusively, expressed in the fruiting body rather than in the mycelium. Most of the assessed basidiomycete mycotoxins have defense‐related functions in the fungi where they occur. However, the exact biological roles and origins of basidiomycete mycotoxins remain an understudied subject.

Although further studies, including more species and structures, are needed to verify the observed phenomenon, the findings presented in this paper offer a promising outlook for expanding the food use of mycelium. They highlight considerable implications for assessing the food safety of new mycelium‐based materials and species, especially as regulatory scrutiny of fungal novel foods is expected to increase in the future.

## Author Contributions

**Niklas Berglund**: writing – original draft, conceptualization, investigation, visualization. **Pauliina Vappu Lankinen**: supervision, writing – review and editing. **Kirsi Susanna Mikkonen**: supervision, project administration, funding acquisition, writing – review and editing.

## Conflicts of Interest

The authors declare no conflicts of interest.
